# Expression of LOX Suggests Poor Prognosis in Gastric Cancer

**DOI:** 10.3389/fmed.2021.718986

**Published:** 2021-09-14

**Authors:** Jinfeng Zhu, Chen Luo, Jiefeng Zhao, Xiaojian Zhu, Kang Lin, Fanqin Bu, Zhonglin Yu, Feilong Zou, Zhengming Zhu

**Affiliations:** Department of Gastrointestinal Surgery, The Second Affiliated Hospital of Nanchang University, Nanchang, China

**Keywords:** gastric cancer, lysyl oxidase, bioinformatics, prognosis, tumor microenvironment

## Abstract

**Background:** Lysyl oxidase (LOX) is a key enzyme for the cross-linking of collagen and elastin in the extracellular matrix. This study evaluated the prognostic role of LOX in gastric cancer (GC) by analyzing the data of The Cancer Genome Atlas (TCGA) and the Gene Expression Omnibus (GEO) dataset.

**Methods:** The Wilcoxon rank-sum test was used to calculate the expression difference of LOX gene in gastric cancer and normal tissues. Western blot and immunohistochemical staining were used to evaluate the expression level of LOX protein in gastric cancer. Kaplan-Meier analysis was used to calculate the survival difference between the high expression group and the low expression group in gastric cancer. The relationship between statistical clinicopathological characteristics and LOX gene expression was analyzed by Wilcoxon or Kruskal-Wallis test and logistic regression. Univariate and multivariate Cox regression analysis was used to find independent risk factors affecting the prognosis of GC patients. Gene set enrichment analysis (GSEA) was used to screen the possible mechanisms of LOX and GC. The CIBERSORT calculation method was used to evaluate the distribution of tumor-infiltrating immune cell (TIC) abundance.

**Results:** LOX is highly expressed in gastric cancer tissues and is significantly related to poor overall survival. Wilcoxon or Kruskal-Wallis test and Logistic regression analysis showed, LOX overexpression is significantly correlated with T-stage progression in gastric cancer. Multivariate Cox regression analysis on TCGA and GEO data found that LOX (all *p* < 0.05) is an independent factor for poor GC prognosis. GSEA showed that high LOX expression is related to ECM receptor interaction, cancer, Hedgehog, TGF-beta, JAK-STAT, MAPK, Wnt, and mTOR signaling pathways. The expression level of LOX affects the immune activity of the tumor microenvironment in gastric cancer.

**Conclusion:** High expression of LOX is a potential molecular indicator for poor prognosis of gastric cancer.

## Introduction

Gastric cancer (GC) is one of the most common gastrointestinal tumors in the world. According to global cancer statistics 2020, there will be more than one million new gastric cancer cases that year, ranking fifth in the global incidence rate (1). Early radical surgical resection is still the only chance to cure gastric cancer. However, even if the margin of early gastric cancer is negative, recurrence of gastric cancer after surgery is still common. Since the diagnosis of GC is usually at an advanced stage, its mortality rate is high, making it the fourth most common cause of cancer-related death (1). Thus, the treatment of gastric cancer is a challenge and requires the use of multiple scientific methods for optimal treatment. In recent years, with the discovery of tumor genetic analysis and tumor markers, targeted therapies in the form of monoclonal antibodies and small-molecule inhibitors have been available; and have become a vital part of GC therapy (2). Therefore, it is particularly important to find a sensitive and specific molecular marker as a target for the treatment of gastric cancer.

Lysyl oxidase (LOX) is a secreted copper-dependent amine oxidase. Its main function is to covalently cross-link collagen and elastic fibers, and reshape the extracellular matrix (ECM) (3). In recent years, more and more evidence has proved that LOX is closely related to the occurrence and development of tumors. The LOX gene is abnormally expressed in a variety of tumors, such as breast cancer, pancreatic cancer, colorectal cancer, hepatocellular carcinoma, and esophageal squamous cell carcinoma (4–8). Meanwhile, LOX is closely related to tumor cell proliferation, invasion, and metastasis (9–11). Studies have reported that LOX is overexpressed in GC (12) and is associated with EMT, invasion, and migration of GC cells (13, 14). However, a past study showed that LOX is a tumor suppressor gene that is inactivated due to methylation and loss of heterozygosity in GC and even other cancers (15). Therefore, the function and prognostic significance of LOX in gastric cancer still need to be further explored.

This study analyzed the data of The Cancer Genome Atlas (TCGA) dataset and the Gene Expression Omnibus (GEO) dataset to evaluate the relationship between LOX gene expression and the clinicopathological characteristics of GC patients and its prognostic significance. It also revealed that LOX may regulate the signaling pathway of gastric cancer, and confirmed that LOX can affect the immune activity of the tumor microenvironment. Provide a more theoretical basis for LOX as a GC biomarker and potential therapeutic target.

## Materials and Methods

### Extraction and Arrangement of TCGA and GEO Data

We have selected the transcriptome profiling and gene expression quantification options of the stomach from TCGA-GDC (https://portal.gdc.cancer.gov/) and downloaded 407 files. The Perl language (http://www.perl.org/) script was used for data sorting, and the original mRNA data of 375 GC tissue samples and 32 corresponding adjacent tissue samples were obtained. The Perl script was used to merge the corresponding gene name and Ensembl ID (http://www.ensembl.org/index.html). Similarly, the clinical data of 443 GC patients were downloaded from TCGA-GDC, and incomplete and duplicated data were deleted, and the clinical data of 316 cases were obtained. Finally, search the GEO database (https://www.ncbi.nlm.nih.gov/geo/) with the keywords “gastric cancer survival” and “Homo sapiens.” Downloaded the data set “GSE84437” with the most samples, and got 433 data sets.

### Tissue Specimens and Immunohistochemical Staining

We collected 40 pairs of GC and para-cancerous tissue specimens in the Second Affiliated Hospital of Nanchang University, and we have obtained the informed consent of the patients in advance. First, the GC and the corresponding adjacent tissues were fixed, paraffin-embedded, sectioned, deparaffinized and hydrated, antigen retrieval, and goat serum blocked. Use anti-LOX (1:100) to incubate overnight at 4°C. Then it was labeled with a secondary antibody for 30 min at room temperature. The specimens were labeled with DAB dye (DAKO) at room temperature for 10 min, and counterstained with Mayer's hematoxylin (DAKO) for 2 min. Finally, take pictures and score. This study was approved by the Medical Research Ethics Committee of the Second Affiliated Hospital of Nanchang University.

### Western Blotting

Use RIPA + PMSF (100:1) to extract total protein from the tissue. After BCA quantification, add appropriate amount of RIPA and Loading buffer to make 2 × loading buffer and boil the total protein. The protein samples were electrophoresed using 10% SDS-PAGE separation gel and transferred to polyvinylidene fluoride (PVDF) membrane. Use 5% skimmed milk to seal the PVDF membrane for 1 h at room temperature. Use the following corresponding antibodies to incubate the PVDF membrane overnight at 4°C: anti-LOX (1:500, 17958-1-AP; Proteintech, Wuhan, China), anti-GAPDH (1:1,0000, 60004-1-Ig; Proteintech, Wuhan, China). The next day, after rinsing 3 times with 1 × TBST, incubate the PVDF membrane with a secondary antibody of the same species at room temperature for 1 h. Next, after washing three times with 1 × TBST, add ECL luminescence reagent for imaging. Finally, Image J software was used to evaluate the relative protein expression level. The relative intensity of the target protein was normalized with an internal control (GAPDH).

### Gene Set Enrichment Analysis

To find the mechanism by which LOX expression affects the prognosis of gastric cancer patients, we explored the signal pathways related to LOX through GSEA (version 3.0). Select the annotated gene set (c2.cp.kegg.v7.2.symbols.gmt) as the reference gene set. By correlating the known functional gene set with the LOX gene expression matrix, we have judged the effect of coordinated changes of genes in this gene set on phenotypic changes. In each analysis, the gene set arrangement was repeated 1,000 times, and the expression level of LOX was regarded as a phenotypic label. The ES calculated for each gene subset is standardized according to the size of the gene set to obtain the Normalized Enrichment Score (NES). Gene sets with NOM *p*-value and FDR *q* < 0.05 are considered to be significantly enriched.

### Statistical Analysis

We used the Wilcoxon rank-sum test to count the differences in the expression of LOX gene in gastric cancer and normal tissues. Kaplan-Meier analysis was used to calculate the survival difference between the high expression group and the low expression group in gastric cancer. We have integrated the original clinical information downloaded by TCGA through Perl language and matched it with LOX expression data. After deleting incomplete clinical information, we obtained 316 clinical data. The relationship between statistical clinicopathological characteristics and LOX gene expression was analyzed by Wilcoxon or Kruskal-Wallis test and logistic regression. Univariate and multivariate Cox regression analysis was used to find independent risk factors affecting the prognosis of GC patients. The CIBERSORT calculation method was used to evaluate the distribution of tumor-infiltrating immune cell (TIC) abundance. All data have been statistically analyzed by R (x64 v.3.5.2). A *p* < 0.05 was considered statistically significant.

## Results

### Overexpression of LOX in GC

**Figure 1:**
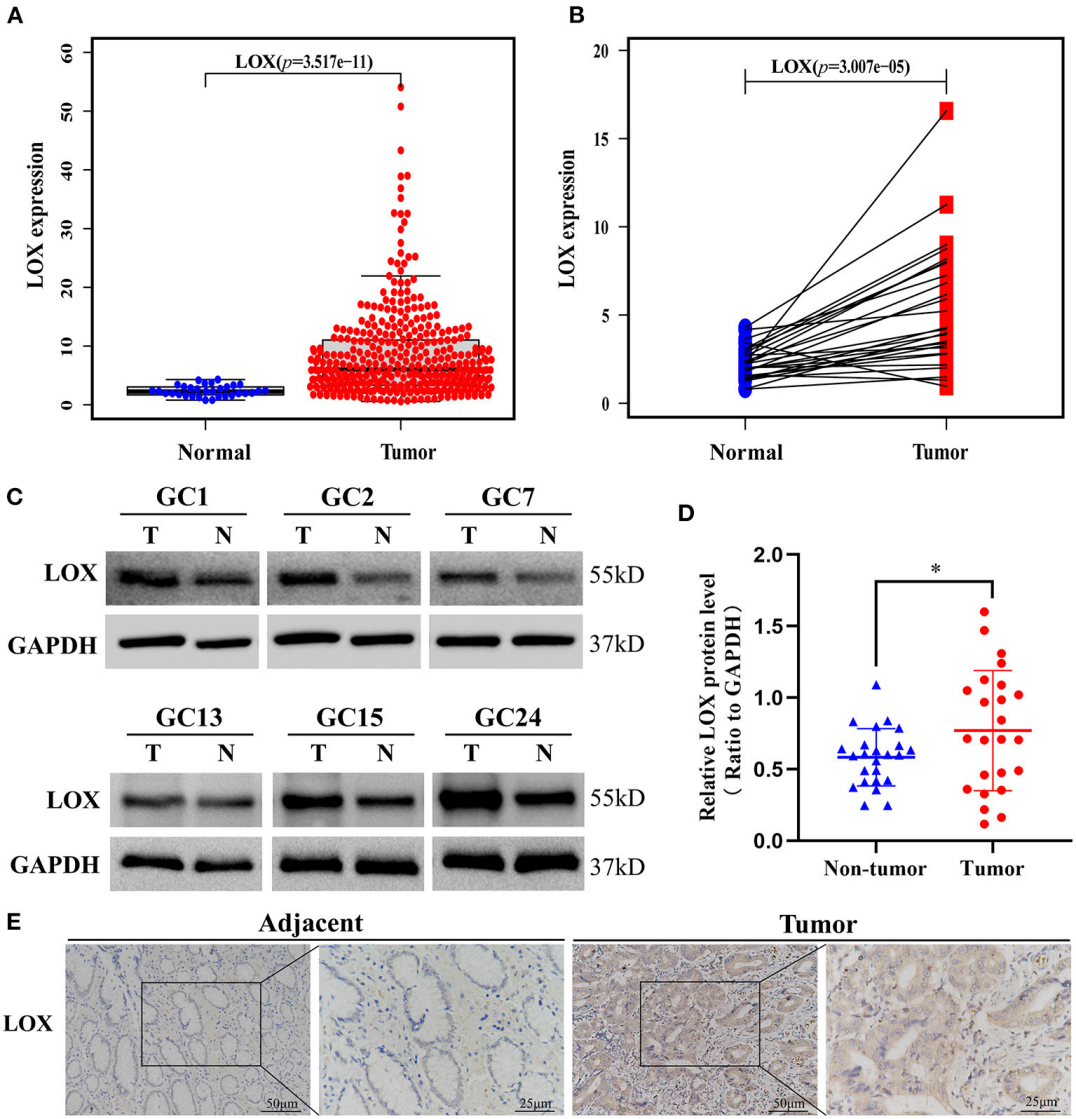
The expression of LOX in GC tissues was significantly higher than that of neighboring normal tissues based on TCGA data. **(A)** The expression of LOX in GC tissues is significantly higher than in normal tissues (*p* = 3.517 × 10^−11^). **(B)** The expression of LOX in GC tissues was significantly higher than that of 27 pairs of non-cancerous adjacent tissues (*p* = 3.007 × 10^−5^). (Wilcoxon rank-sum test). **(C,D)** The protein expression levels of LOX in GC tissues and matched non-tumor tissues were evaluated by Western blot (*p* = 0.0189; *n* = 24; T, tumor; NT, non-tumor). **(E)** LOX representative IHC stained images in GC tissue and adjacent tissues (*n* = 40; magnification: left, 200×; right, 400×). TCGA, The Cancer Genome Atlas. *p < 0.05.

We obtained LOX mRNA expression from the information of 375 GC tissue samples and 32 normal tissue samples downloaded by TCGA. The scatter plot shows that compared with normal tissues, the expression of LOX mRNA in GC tissues was significantly increased (*p* = 3.517 × 10^−11^; Figure 1A). Subsequently, we found 27 pairs of GC tissues and corresponding adjacent samples from the information of 407 samples. Through Wilcoxon rank-sum test, it was found that the expression of LOX in GC tissue was significantly higher than the corresponding adjacent tissues (*p* = 3.007 × 10^−5^; Figure 1B). We found through Western blotting that compared with the corresponding adjacent tissues, LOX protein was highly expressed in gastric cancer tissues (*p* = 0.0189; Figures 1C,D). Similarly, we found through IHC that LOX protein was highly expressed in gastric cancer (Figure 1E). In summary, we can conclude that LOX mRNA and protein are highly expressed in gastric cancer.

### LOX Over-Expression Is Associated With Poor Survival in GC

**Figure 2:**
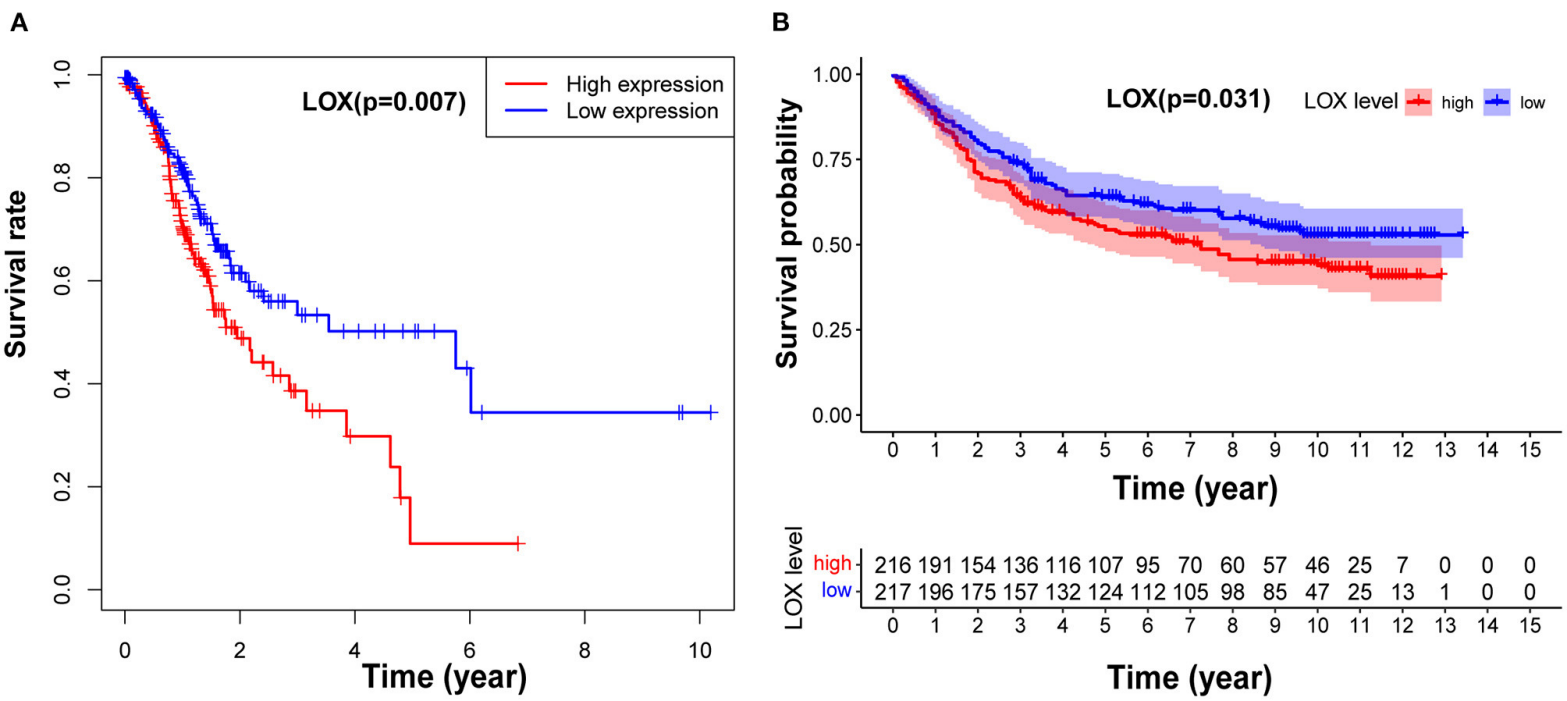
LOX impact on the overall survival of patients with GC. **(A)** Kaplan-Meier curve was used to analyze the relationship between LOX mRNA expression and prognosis of GC patients based on TCGA data (*p* = 0.007). **(B)** Kaplan-Meier curve was used to analyze the relationship between LOX mRNA expression and prognosis of GC patients based on GEO data (*p* = 0.031). TCGA, The Cancer Genome Atlas; GEO, the Gene Expression Omnibus.

To clarify the relationship between LOX and GC prognosis. First, we used the Kaplan-Meier risk estimation method to assess the difference in survival rates between high and low expression of LOX in GC patients in TCGA. The results showed that the survival rate of the high LOX expression group was significantly lower than that of the low LOX expression group, and the difference was statistically significant (*p* = 0.007; Figure 2A). Subsequently, we repeatedly verified the clinical information of 433 cases extracted from the GEO database. Similarly, we found that compared with the low LOX expression group, the survival rate of GC patients in the high LOX expression group was worse (*p* = 0.031; Figure 2B). In summary, we can conclude that LOX is highly expressed in GC and is significantly related to poor survival.

### Association Between LOX and Clinicopathological Characteristics of GC Patients

**Figure 3:**
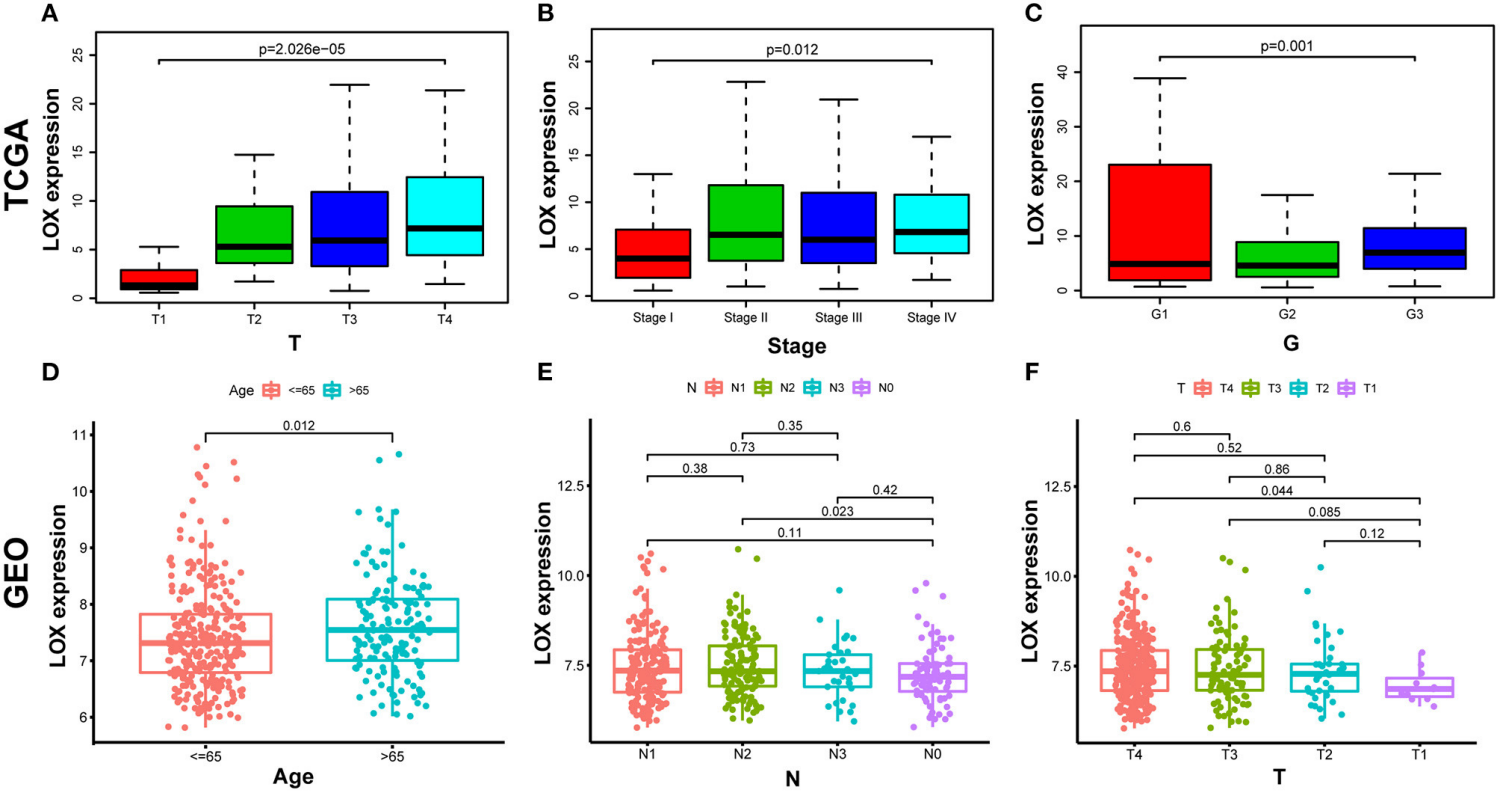
The relationship between LOX expression and clinicopathological characteristics. **(A–C)** Analyze the relationship between LOX expression and T stage, clinical-stage, and histologic grade based on TCGA data. **(D–F)** Analyze the relationship between LOX expression and Age, T stage and N stage based on GEO data. (Wilcoxon or Kruskal-Wallis test). TCGA, The Cancer Genome Atlas; GEO, the Gene Expression Omnibus.

We compiled the data of 422 cases extracted from TCGA, and after removing incomplete information, we obtained sample information of 316 cases (Table 1). Wilcoxon or Kruskal-Wallis test showed that: high LOX expression is significantly related to T stage (*p* = 0.001), clinical-stage (*p* = 0.012), and histologic grade (*p* = 2.026 × 10^−5^; Figures 3A–C). In the same way, we repeated verification through 433 cases of data collected by GEO. The results showed that the up-regulation of LOX in GC was significantly correlated with T classification (T4 vs. T1), age, and N classification (N2 vs. N0) (all *p* < 0.05; Figures 3D–F). In addition, we grouped based on the median value of LOX expression and performed logistic regression analysis. The data shows that LOX overexpression in GC is related to high grade (OR = 1.98 for G3 vs. G2) and T classification (OR = 9.21 for T4 vs. T1; OR = 6.25 for T3 vs. T1; OR = 6.30 for T2 vs. T1) (all *p* < 0.05) is significantly correlated (Table 2). Based on the above results, we found that the high expression of LOX is significantly correlated with T-stage progression in gastric cancer. The above evidence shows that compared with GC patients with low LOX expression, GC with high LOX expression is more malignant.

**Table 1:** Clinical characteristics of patients with gastric cancer.

	***n***	**Proportion (%)**
**Age**		
≤ 65 years	140	44.30
>65 years	176	55.70
**Sex**		
Male	196	62.03
Female	120	37.97
**Histological grade**		
G1	7	2.22
G2	108	34.18
G3	201	63.61
**Stage**		
I	42	13.29
II	101	31.96
III	138	43.67
IV	35	11.08
**T classification**		
T1	15	4.75
T2	63	19.94
T3	151	47.78
T4	87	27.53
**M classification**		
M0	294	93.04
M1	22	6.96
**N classification**		
N0	99	31.33
N1	83	26.27
N2	69	21.84
N3	65	20.57
**Survival status**		
Death	111	35.13
Survival	205	64.87

**Table 2:** LOX expression associated with clinical characteristics (logistic regression).

**Clinical characteristics**	**Total (*n*)**	**The odd ratio in LOX expression**	***p*** **-value**
Age (>65 vs. ≤ 65 years)	316	1.05 (0.68–1.64)	0.821
Sex(Male vs. Female)	316	1.18 (0.75–1.85)	0.487
**Histological grade**			
(G3 vs. G1)	209	1.68 (0.36-8.70)	0.505
(G2 vs. G1)	115	0.88 (0.19–4.66)	0.873
(G3 vs. G2)	309	1.98 (1.23–3.20)	**0.005**
**Stage**			
(IV vs. I)	77	2.17 (0.88–5.50)	0.460
(III vs. I)	180	1.63 (0.81–3.35)	0.690
(II vs. I)	143	1.79 (0.87–3.80)	0.989
**T classification**			
(T4 vs. T1)	102	9.21 (2.36–61.22)	**0.004**
(T3 vs. T1)	166	6.25 (1.65–40.83)	**0.018**
(T2 vs. T1)	78	6.30 (1.57–42.41)	**0.021**
**M classification**			
**(M1 vs. M0)**	316	1.48 (0.62–3.70)	0.379
**N classification**			
(N3 vs. N0)	164	1.27 (0.68–2.38)	0.461
(N2 vs. N0)	168	0.88 (0.48–1.63)	0.691
(N1 vs. N0)	182	1.00 (0.56–1.79)	0.989

*Bold values indicate p < 0.05*.

### LOX Overexpression Was an Independent Factor for Poor Prognosis of GC

**Figure 4:**
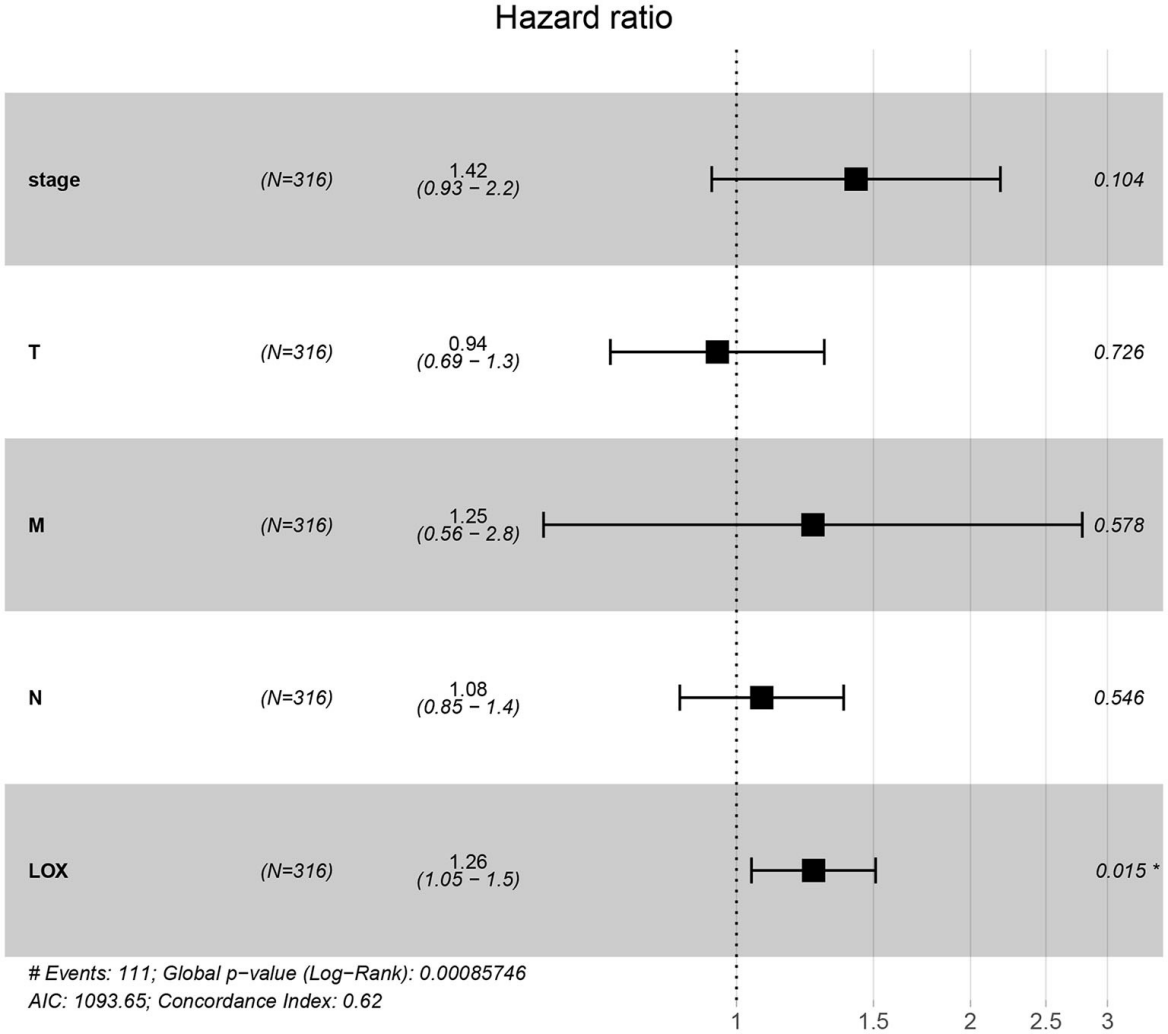
Forest plot of the correlation between LOX expression and overall survival in GC patients based on TCGA data. TCGA, The Cancer Genome Atlas.

**Figure 5:**
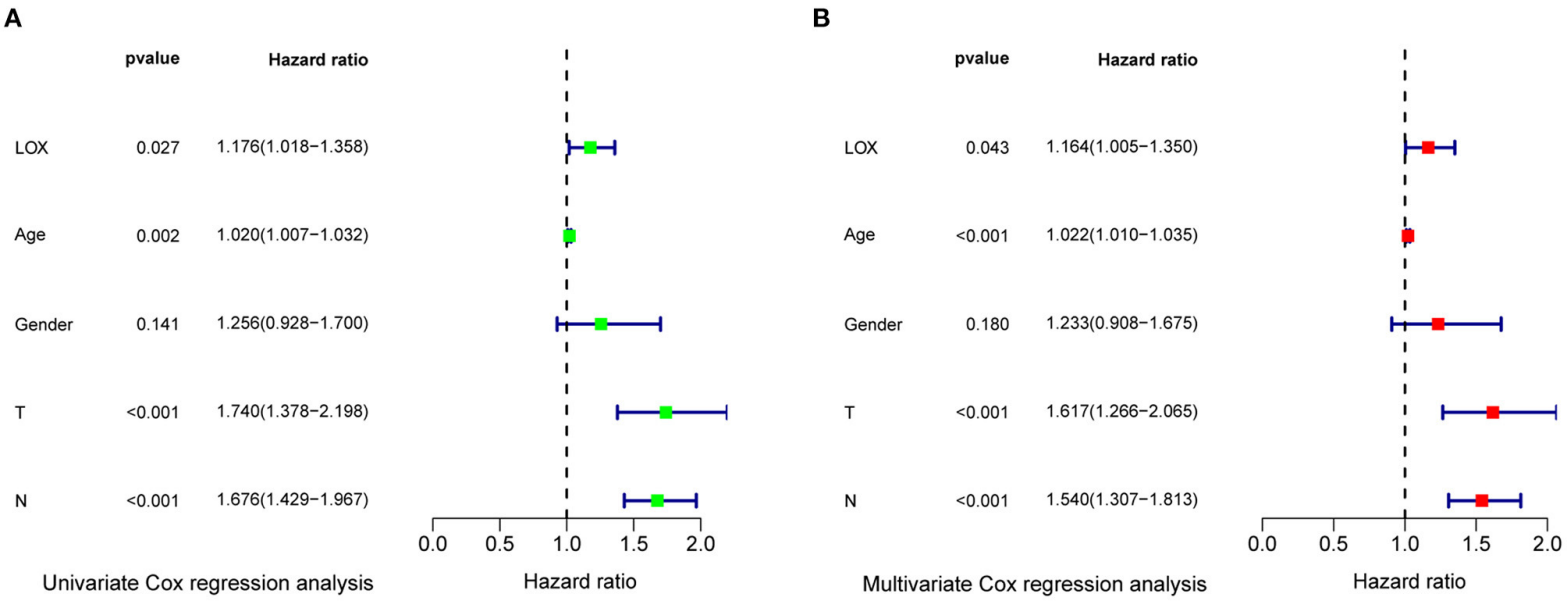
Forest plot of the correlation between LOX expression and overall survival in GC patients based on GEO data. **(A)** Univariate Cox regression analysis. **(B)** Multivariate Cox regression analysis. GEO, the Gene Expression Omnibus.

To evaluate the impact of high LOX expression and other clinicopathological characteristics on survival, we conducted univariate and multivariate Cox regression analyses. First, we analyze the complete information of 316 cases from TCGA. Univariate Cox regression analysis shows that the factors affecting the poor prognosis of gastric cancer are age (*p* = 0.008114), stage (*p* = 0.000209), T classification (*p* = 0.031105), M classification (*p* = 0.026161), N classification (*p* = 0.004116), and LOX(*p* = 0.043712; Table 3). Multivariate Cox regression analysis found that LOX (*p* = 0.014948) is an independent factor of the poor prognosis of GC (Figure 4 and Table 3). Furthermore, we repeated the verification of 433 cases of GEO information. Univariate Cox regression analysis pointed out that age, T, N, and LOX (all *p* < 0.05) are all factors affecting the prognosis of gastric cancer (Figure 5A). Multivariate Cox regression analysis yielded the same results as above (Figure 5B).

**Table 3:** Univariate analysis and multivariate analysis of the correlation of LOX expression with overall survival among patients with gastric cancer.

**Univariate cox regression analysis**					**Multivariate cox regression analysis**				
**Id**	**HR**	**HR.95L**	**HR.95H**	***p*** **-value**	**Id**	**HR**	**HR.95L**	**HR.95H**	***p*** **-value**
Age	1.025929	1.006668	1.045557	**0.008114**					
Gender	1.500531	0.990844	2.272401	0.055298					
Grade	1.385611	0.958427	2.003196	0.082886					
Stage	1.53973	1.225594	1.934384	**0.000209**	Stage	1.424875	0.92932	2.184682	0.104417
T	1.297104	1.023904	1.6432	**0.031105**	T	0.944841	0.688346	1.296913	0.725507
M	2.032579	1.087801	3.797917	**0.026161**	M	1.253902	0.564725	2.784136	0.57825
N	1.284776	1.082644	1.524648	**0.004116**	N	1.077589	0.8454	1.373549	0.546152
LOX	1.021463	1.000599	1.042763	**0.043712**	LOX	1.256338	1.045416	1.509815	**0.014948**

*Bold values indicate p < 0.05*.

### GSEA Identifies the Relevant Signal Pathways of LOX

**Figure 6:**
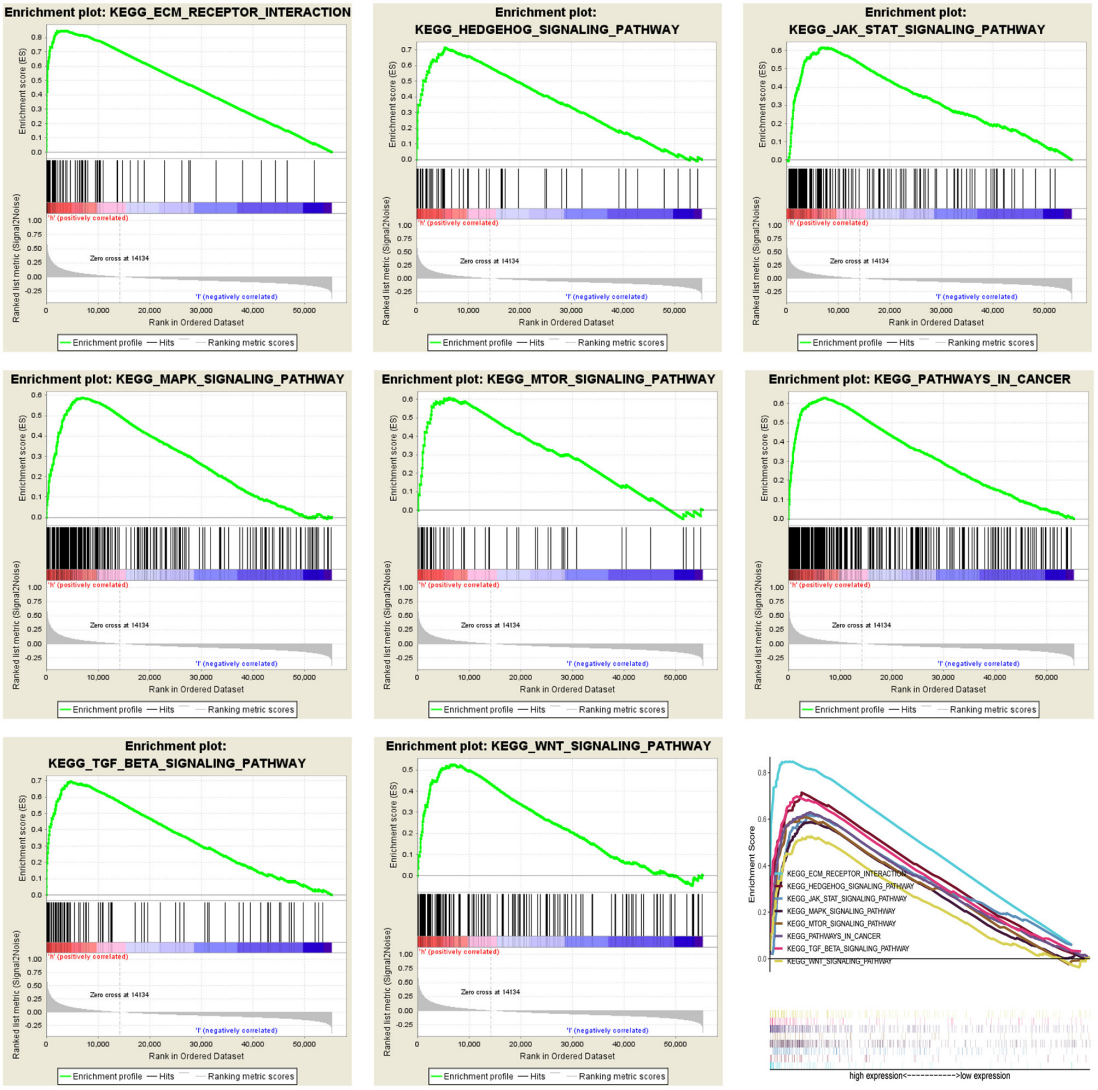
Merged enrichment maps from gene set enrichment analysis (GSEA), including enrichment scores and gene sets. Significantly enriched signaling pathways include ECM-receptor-interaction, pathways in cancer, Hedgehog signaling, TGF-beta signaling, JAK-STAT signaling, MAPK signaling, Wnt signaling, mTOR signaling.

To determine the potential mechanism of LOX expression affecting the prognosis of gastric cancer patients, we performed gene enrichment analysis. Based on the filtering of NES, NOM *p*-value, and FDR *q*-value, a significantly rich signal pathway was selected. The results showed that 8 signaling pathways involved in ECM- receptor-interaction, pathways in cancer, Hedgehog signaling, TGF-beta signaling, JAK-STAT signaling, MAPK signaling, Wnt signaling, mTOR signaling were differentially enriched in the highly expressed phenotypes of LOX (Figure 6 and Table 4).

**Table 4:** Gene sets enriched in the high LOX expression phenotype.

**Name**	**ES**	**NES**	**NOM *p*-val**	**FDR *q*-val**
KEGG_ECM_RECEPTOR_INTERACTION	0.847	2.496	0.000	0.000
KEGG_PATHWAYS_IN_CANCER	0.629	2.375	0.000	0.000
KEGG_HEDGEHOG_SIGNALING_PATHWAY	0.714	2.368	0.000	0.000
KEGG_TGF_BETA_SIGNALING_PATHWAY	0.698	2.331	0.000	0.000
KEGG_JAK_STAT_SIGNALING_PATHWAY	0.617	2.299	0.000	0.000
KEGG_MAPK_SIGNALING_PATHWAY	0.588	2.291	0.000	0.000
KEGG_WNT_SIGNALING_PATHWAY	0.526	1.937	0.000	0.005
KEGG_MTOR_SIGNALING_PATHWAY	0.609	2.020	0.002	0.002

*NES, normalized enrichment score; NOM, nominal; FDR, false discovery rate. Gene sets with NOM p < 0.05 and FDR q < 0.05 are considered as significant*.

### Correlation Between LOX Expression and TIC Ratio in Gastric Cancer

**Figure 7:**
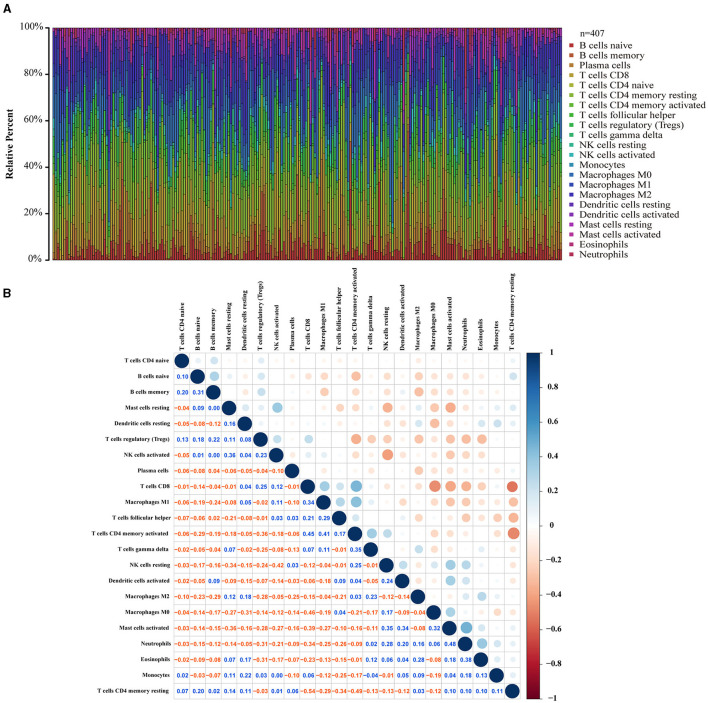
TIC spectrum and correlation analysis in GC samples. **(A)** The bar graph shows the proportion of 22 TICs in the GC. **(B)** The heat map shows the correlation between 22 TICs (Pearson coefficient test). The number represents the *p*-value corresponding to the correlation between the two immune cells. The color depth of each box represents the corresponding correlation value between the two types of immune cells.

**Figure 8:**
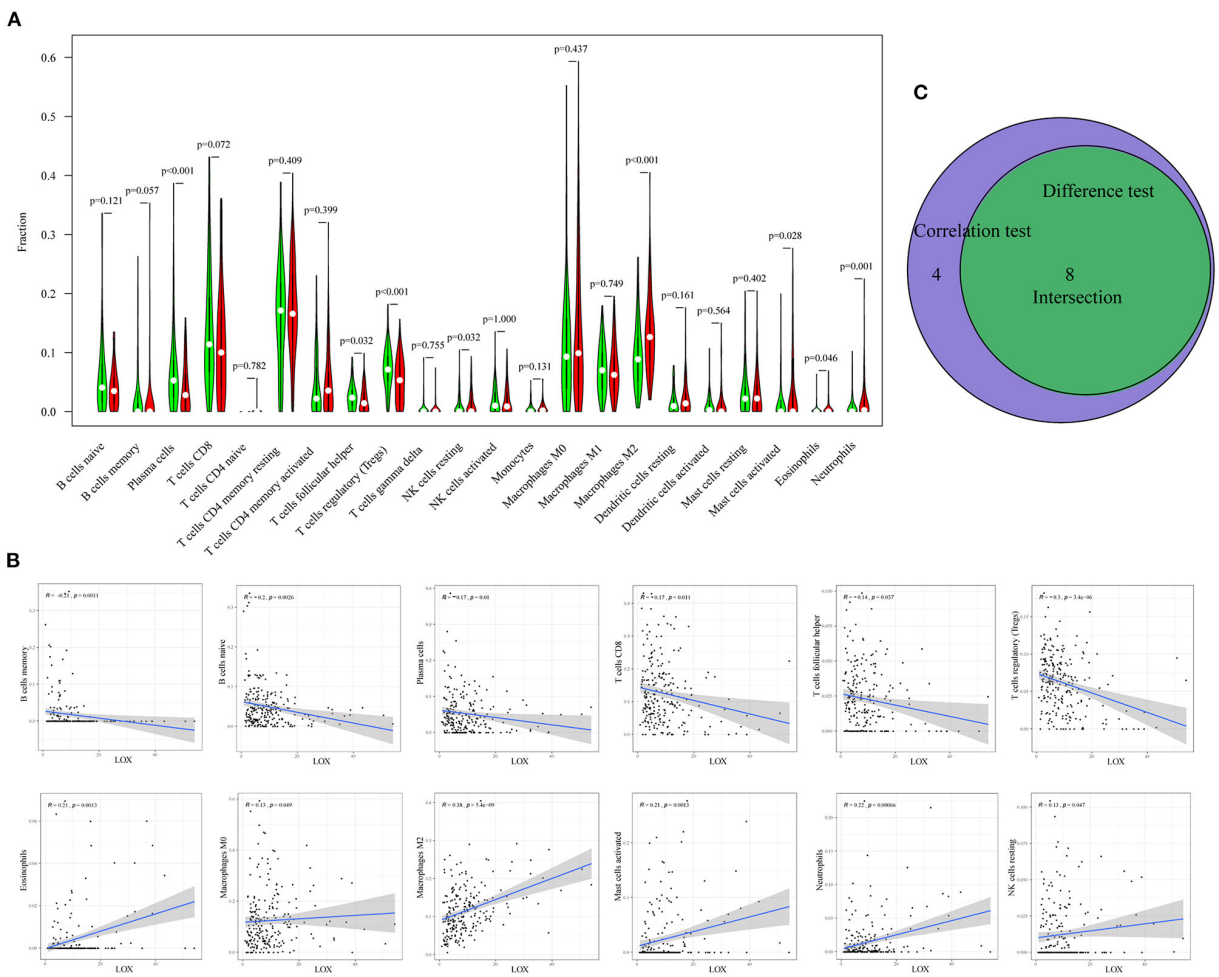
Correlation between TIC ratio and LOX expression. **(A)** The violin chart shows the proportional difference between the LOX high and low expression groups and 22 TICs (Wilcoxon rank-sum test). **(B)** The Scatter diagram shows the correlation between LOX expression and the proportion of TIC in 12 (Pearson coefficient test). **(C)** Venn diagram shows that 8 TICs are related to LOX expression. The intersection of the difference and correlation test is shown in the violin chart and the scatter chart.

To clarify the correlation between LOX expression and the immune microenvironment, we used the CIBERSORT method to analyze the proportion of 22 immune cell subpopulations in tumor infiltration (Figure 7). The analysis results of differences (Figure 8A) and correlation (Figure 8B) have indicated that there are 8 TICs related to LOX expression (Figure 8C). The five positive correlations were NK cells resting, Macrophages M2, Mast cells activated, Eosinophils, and Neutrophils. The three negative correlations were Plasma cells, T cells follicular helper, and T cells regulatory (Tregs). These results indicate that the expression level of LOX affects the immune activity of gastric cancer TEM.

## Discussion

The lysyl oxidase (LOX) family consists of LOX and four LOX-like proteins (LOXL1, LOXL2, LOXL3, and LOXL4) (16). Studies have found that the abnormal expression of LOX family proteins is usually associated with a variety of diseases related to ECM changes, such as fibrotic eosinophilic esophagitis (17), primary myelofibrosis (18), Wilson's disease (19), thoracic aortic aneurysm and dissection (20), fibrosis in systemic sclerosis (21), and human tumors. Studies have reported that the expression of LOX family members LOXL1, LOXL3, and LOXL4 is related to distant metastasis of gastric cancer (22). Therefore, the influence of LOX expression on GC deserves further discussion. LOX is located on chromosome 5q23.1 and is a key enzyme for the cross-linking of collagen and elastin in the extracellular matrix. Overexpression of LOX plays an important role in extracellular matrix remodeling and angiogenesis in various cancers. Previous studies reported that high LOX expression is associated with poor survival of triple-negative breast cancer, and silencing LOX can induce breast cancer cell apoptosis and reduce its chemotherapy resistance (4). Natarajan et al. showed that increased expression of LOX promotes collagen cross-linking and tumor cell invasion, and induces peritoneal metastasis of high-grade serous ovarian cancer (HGSOC) (23). Kasashima et al. have demonstrated that LOX knockdown can inhibit hypoxia-induced epithelial-mesenchymal transition (EMT) of GC cells (13). Zhu et al. pointed out that knocking down LOX attenuates the proliferation, migration, and invasion of HCC cells, and reduces the expression of vascular endothelial growth factor (VEGF) through the p38 mitogen-activated protein kinase (MAPK) signaling pathway (24). Wei et al. demonstrated that P-selectin-mediated platelet aggregation may up-regulate LOX expression and enhance the ECM of remodeling and hardening tumors, which may promote the progression of colorectal cancer (6). In addition, LOX silence inhibits hypoxia-mediated non-small cell lung cancer (NSCLC) cell invasion and migration (25). Similarly, knocking out LOX and inhibiting LOX activity in Anaplastic Thyroid Cancer (ATC) cells can significantly reduce cell invasion, migration and metastasis *in vitro* and *in vivo* (26). Conversely, the upregulation of LOX increased the proliferation capacity and induced angiogenesis in oral squamous cell carcinoma (OSCC) cells (27). As mentioned in the above studies, high LOX mRNA and protein expression play a key role in promoting many types of cancer.

Interestingly, LOX can also act as a tumor suppressor (28). Bouez et al. reported that it is the LOX propeptide (LOX-PP) that exerts a tumor suppressor effect (29), regardless of its enzyme activity (30). Similarly, Min et al. reported that LOX-PP is a tumor suppressor in the population of African American triple-negative breast cancer patients (31). Kaneda et al. proved that LOX has an anti-tumor effect in human GC cells, but the molecular mechanism of how LOX exerts its anti-tumor activity is not involved (15). Kaneda et al.'s experiment may still have some imperfect aspects, such as only overexpression of LOX without silence; human GC cell line only uses MKN28 and KATO III; *in vivo* experiments only uses MKN28 cell line, etc. However, there is strong evidence that the mature form of LOX plays a key role in remodeling the extracellular matrix and promotes tumor development and metastasis (32, 33).

Recently, many studies have shown LOX as a potential prognostic marker. Umezaki et al. found that high LOX expression is related to EMT markers, which can predict early recurrence and poor survival of HCC patients. This suggests that LOX may be a potential therapeutic target for early recurrence of HCC (7). In addition, Johnston et al. proved that high LOX expression is associated with poor prognosis and EGFR phosphorylation in patients with lung cancer (34). Similarly, Yu et al. pointed out that LOX mRNA is overexpressed in OSCC samples, and the high expression of LOX is significantly associated with aggressive clinicopathological characteristics, overall survival, and disease-free survival. Therefore, LOX can be used as an independent prognostic predictor of OSCC patients (27). In a biomimetic 3D model technology of hypoxia-driven cancer progression, it was found that LOX is the most up-regulated marker in the model, and LOX has prognostic significance for breast cancer patients (35). Similar findings were previously found in high-grade serous ovarian cancer (HGSOC) (36), gastric cancer (37), colorectal cancer (38), and pancreatic cancer (5). Consistent with these findings, our results show that LOX is significantly up-regulated in GC tissues compared to paired normal tissues. In addition, the increase of LOX expression in GC tissues is related to the advanced T stage and poor overall survival rate. This indicates that LOX is closely related to the degree of malignancy of GC, and LOX overexpression can predict a poor prognosis of GC. COX regression analysis showed that LOX overexpression is an independent prognostic factor of GC, which indicates that LOX may be a potential biomarker affecting the prognosis of GC.

In this study, we found that the high expression of LOX is related to the ECM receptor interaction, cancer, Hedgehog, TGF-beta, JAK-STAT, MAPK, Wnt, and mTOR signaling pathways of GSEA. It has been reported that these signaling pathways are related to the occurrence, development and malignant phenotype of GC. For example, CD44v6 overexpression enhances the malignancy of GC cells by regulating ECM mediated by adipose stromal cells (39). Research by Lourenco et al. showed that SPOP inhibits the occurrence of GC by regulating the Hedgehog/Gli2 signaling pathway (40). Zeng et al. pointed out that RHBDF2 induces the invasion ability of GC cells by activating TGF-beta1 Signaling (41). Ishimoto et al. found that the inhibition of GC cell proliferation by SOCS-1 is mediated through JAK-STAT and p38 MAPK signaling pathways (42). In addition, RACK1 suppresses gastric tumorigenesis by stabilizing the β-catenin destruction complex (43). Therefore, the above research results indicate that LOX promotes GC cell growth and poor overall survival rate may be through the above signaling pathway. Thus, LOX can be used as a new treatment for GC Targets and prognostic molecules.

With the in-depth study of tumor biology, more and more attention is paid to the role of environmental conditions around cancer cells. Tumor microenvironment (TME) refers to the surrounding micro-environment where tumor cells exist, including surrounding blood vessels, immune cells, fibroblasts, bone marrow-derived inflammatory cells, various signaling molecules, and extracellular matrix (ECM) (44). In various tumors, overexpression of LOX induced by a hypoxic environment plays an important role in ECM remodeling and angiogenesis. Many institutions have focused on the development of targeted LOX inhibitors and nano-therapies. Saatci et al. demonstrated that targeting LOX can overcome chemotherapy resistance in triple-negative breast cancer (4). De Vita et al. reported: Lysyl oxidase engineered lipid nanovesicles for the treatment of triple-negative breast cancer (45). Targeting LOX family-mediated matrix cross-linking as an anti-matrix therapy for solid tumors (46). Similarly, Setargew et al. found that radioactive LOX-trackable nanoparticles (LOX ab-NPs) can be used as chemotherapy carriers for combined targeted radiotherapy and chemotherapy in clinical oncology (47). However, most patients cannot benefit from currently available immunotherapies, and many patients will experience immune-related adverse events. TME is the main reason for the failure of nanomedicine and immunotherapy (48). Research on TME may improve the efficacy of current therapies and provide new opportunities for therapeutic targeting. Therefore, standardizing TME for immunotherapy will promote the delivery of nanomedicine and reduce immunosuppression in TME (48). For example, blocking integrin signaling and its downstream tyrosine kinase signaling will improve the efficacy of radiotherapy and molecular targeted therapy (49). In this study, we found that LOX expression may be involved in the regulation of immune cell function and the conversion of metabolic state. According to the analysis of the TIC ratio by the CIBERSORT algorithm, we found that with the increase of LOX, the ratio of NK cells resting, Macrophages M2, and Mast cells activated will also increase. In TME, tumor-associated macrophages (TAM), and Mast cells activated secrete a variety of cytokines to stimulate tumor cell proliferation and angiogenesis, which are related to tumor progression, metastasis, and prognosis (50, 51). On the contrary, the proportion of Plasma cells, T cells follicular helper, and T cells regulatory (Tregs) decreased accordingly. This indicates that the immune function against GC has decreased. Thus, we can speculate that LOX may be an indicator of TME state transition. Therefore, when we target LOX therapy, we also need to consider its role in TEM.

However, our research still has limitations. First, most of the data comes from public databases. In order to avoid the analysis bias caused by the current retrospective research, we will continue to conduct forward-looking research in the future. Secondly, the current research data is high-throughput gene sequencing data derived from databases, and it is impossible to clearly assess the direct mechanism of LOX's involvement in the development of GC. Therefore, further *in vivo* and *in vitro* experimental studies are necessary. Finally, our study did not include cases of neoadjuvant chemotherapy or radiotherapy for analysis, and the exploration of LOX function is not perfect. Therefore, we will continue to work hard to explore and improve the role of LOX on GC.

## Data Availability Statement

Publicly available datasets were analyzed in this study, these can be found in The Cancer Genome Atlas (https://portal.gdc.cancer.gov/); the NCBI Gene Expression Omnibus (GSE84437).

## Ethics Statement

The studies involving human participants were reviewed and approved by Medical Research Ethics Committee of the Second Affiliated Hospital of Nanchang University Nanchang University. The patients/participants provided their written informed consent to participate in this study.

## Author Contributions

ZZ: had full access to all of the data in the study, takes responsibility for the integrity of the data, and the accuracy of the data analysis. JZhu, CL, and ZZ: study concept and design. JZhu: drafting of the manuscript. XZ, KL, and FB: statistical analysis. ZZ: obtained funding and study supervision. All authors acquisition, analysis, or interpretation of data, critical revision of the manuscript for important intellectual content, and administrative, technical, or material support.

## Funding

This study was funded by the National Natural Science Foundation of China (No. 881560389), the Project of the Jiangxi Provincial Department of Science and Technology (Nos. 20181BBG70015 and 20202BABL206091), and the Jiangxi Provincial Health Department (No. 20171068).

## Conflict of Interest

The authors declare that the research was conducted in the absence of any commercial or financial relationships that could be construed as a potential conflict of interest.

## Publisher's Note

All claims expressed in this article are solely those of the authors and do not necessarily represent those of their affiliated organizations, or those of the publisher, the editors and the reviewers. Any product that may be evaluated in this article, or claim that may be made by its manufacturer, is not guaranteed or endorsed by the publisher.
